# Cytokine response patterns to complex biofilms by mononuclear cells discriminate patient disease status and biofilm dysbiosis

**DOI:** 10.1080/20002297.2017.1330645

**Published:** 2017-06-12

**Authors:** I. M. Velsko, Y. Cruz-Almeida, H. Huang, S. M. Wallet, L. M. Shaddox

**Affiliations:** ^a^ Department of Periodontology, College of Dentistry, University of Florida, Gainesville, FL, USA; ^b^ Institute for Aging, College of Medicine, University of Florida, Gainesville, FL, USA; ^c^ Department of Oral Biology, College of Dentistry, University of Florida, Gainesville, FL, USA

**Keywords:** Periodontal disease, localized aggressive periodontitis, subgingival biofilm, host–pathogen interaction, inflammatory response, biofilm homeostasis, cytokine

## Abstract

Localized aggressive periodontitis (LAP) is a rare form of periodontal disease with site-specific rapid tissue destruction. A lipopolysaccharide (LPS) hyper-inflammatory response was shown in LAP using peripheral whole blood, although responses to other bacterial surface components or complex oral biofilms have not been evaluated. Peripheral blood mononuclear cells (PBMCs) from 14 LAP patients, 15 healthy siblings (HS), and 13 unrelated healthy controls (HC) were stimulated with: LPS, lipoteichoic acid, or peptidoglycan; intact or sonically dispersed *in vitro*–grown biofilms from a LAP disease site, a LAP healthy site, or a healthy control site. Cell culture supernatants were assayed for 14 cyto/chemokines. Discriminant function analysis determined cyto/chemokines that discriminate disease status by response patterns to different stimuli. Qualitative differences in the cytokine response pattern among patient groups were observed to intact and dispersed biofilms, yet responses to healthy and diseased biofilms could not be discriminated. Despite an equivalent magnitude of response, LAP-derived PBMCs demonstrated a qualitatively different pattern of response to LPS and dispersed biofilms. PMBCs from each group responded distinctly to stimulation withsubgingival biofilms. Multiple underlying mechanisms related to bacterial-induced inflammatory responses can culminate in LAP disease initiation and/or progression, and biofilm homeostasis could play an important role.

## Introduction

Localized aggressive periodontitis (LAP), formerly known as localized juvenile periodontitis or localized early-onset periodontitis, is a rare, rapidly progressing form of periodontal disease affecting children and young adults. LAP manifests as significant periodontal ligament and alveolar bone destruction affecting primarily first molars and incisors [1]. Despite severe tissue destruction, there is relatively little clinical inflammation and low calculus burden at affected sites compared to chronic periodontitis [2]. It was previously reported that whole blood from LAP patients demonstrated a hyper-responsiveness to LPS stimulation, which was attributed to monocyte over-activation by TLR signaling [3]. This finding is supported by prior publications that demonstrated an LPS-induced hyper-response in LAP patient adherent mononuclear cells [4,5].

While the trigger for LAP development is not completely understood, the reported immune hyper-responsivness suggests that immune cells entering the periodontal tissues may hyper-respond to bacteria or their components in the periodontal pocket, and drive significant local inflammation, resulting in soft-tissue and bone destruction. Circulating monocytes and their tissue-counterpart macrophages play a substantial role in driving periodontal inflammation by contributing to all aspects of tissue destruction. Initially, production of cyto/chemokines by these cells as a consequence of innate immune receptor signaling can recruit additional immune cells to the periodontium [6,7]. Phagocytosis of microorganisms and presentation of antigens results in activation of adaptive immunity, which in turn augments innate immune responses [7]. In addition, macrophages can produce matrix metalloproteinases (MMPs), which can also directly contribute to periodontal tissue destruction [6,7], while the cytokines produced by macrophages can stimulate other local cell types to produce similar MMPs [7]. Finally, macrophages are also involved in initiating osteoclast-mediated bone resorption by production of cytokines such as interleukin 1 beta (IL-1β) that promote osteoclast activation [7].

Lipopolysaccharide (LPS) initiates inflammatory signaling by binding to innate immune receptors such as Toll-like receptor 4 (TLR4) on host cell surfaces, which activates a signaling cascade that results in increased production of inflammatory cytokines and chemokines. However, there are numerous bacterial surface components in addition to Gram-negative bacterial LPS that can incite similar innate immune signaling. It is likely that innate immune cells in the periodontal tissues encounter a wide variety of bacterial products because of the complex polymicrobial nature of the subgingival biofilm. One such likely abundant stimulant is Gram-positive bacterial lipoteichoic acid (LTA), a long-chain carbohydrate with a lipid anchor that is exposed on the bacterial surface. Indeed, LTA is considered the main inflammatory component responsible for Gram-positive septic shock [8,9], whereby it has been shown to stimulate cytokine production from human peripheral blood mononuclear cells (PBMCs) and human umbilical vein endothelial cells at levels comparable to that of LPS stimulation at similar concentrations [10,11]. Although LTA is primary recognized by TLR2 rather than TLR4, the inflammatory signaling pathway initiates the production of similar groups of inflammatory cytokines [12]. Additionally, peptidoglycan (PGN), the molecule that comprises both Gram-positive and -negative bacterial cell walls, is known to activate inflammatory signaling in myriad cell types [13]. Gram-positive bacteria have much more PGN than Gram-negative bacteria and release a much greater percentage of it during growth and restructuring [14], making it likely that PGN encountered by host immune cells is largely Gram positive in origin. While synthetic PGN fragments are known to activate intracellular innate immune receptors NOD1 and NOD2 [15,16], the extracellular receptor has not been definitively determined [14].

Although LPS, LTA, and PGN are well-characterized bacterial immunostimulants, complex oral biofilms contain numerous other microbial components that may both stimulate and inhibit host inflammatory reactions [17]. In fact, influenza virus infection of primary macrophages elicits substantially different gene expression patterns than does stimulation of the same cells with individual TLR stimulants [18]. Such distinct responses highlight the necessity of studying how complex infections elicit immune responses. In addition, biofilms with differing microbial composition may present with a different spectrum and/or concentration of immunostimulants. Significant differences in the microbiota inhabiting LAP diseased and periodontally healthy sites have been reported [19,20]. Yet, it is not clear if these differences are a cause of or a consequence of disease, or whether the composition of each of these biofilms differentially influence the local immune response. It has previously been determined that the cytokine response of whole blood from LAP patients to dispersed subgingival plaque samples from either a periodontally healthy or diseased site is equivalent in magnitude [21], which may suggest that the microbial biofilm composition may not be an important factor in eliciting the immune hyper-responsiveness that is observed.

However, monocyte interaction with intact biofilms may be distinct from interactions with dispersed biofilm-grown bacteria. Indeed, there is evidence that macrophage responses to intact biofilms versus mechanically disrupted biofilms or planktonically grown bacteria differ for individual species grown in monoculture [22–24]. However, these macrophage responses were not from patients with inflammatory disorders, and it is possible that there are differences in the way monocytes from healthy and inflammatory disease backgrounds recognize and respond to biofilms.

This study investigated the PBMC response of LAP patients, their healthy siblings, and unrelated healthy controls to stimulation with LPS, LTA, and PGN, as well as with intact and dispersed biofilms from periodontally healthy and diseased sites. The study demonstrates that cytokine responses differ qualitatively based on stimulant, such that this pattern of reactivity can be used to discriminate LAP disease cohorts from healthy cohorts, potentially offering new ways to diagnose and treat patients.

## Materials and methods

### Participant population

Participants were recruited from Leon and Duval County Health Departments in Tallahassee and Jacksonville, FL, respectively, and from the dental clinics at University of Florida, Gainesville, FL, between March 2015 and August 2015. All participants or their legal representatives signed an informed consent form approved by the University of Florida Institutional Review Board. LAP patients fulfilled the following inclusion criteria: 5–25 years old, having at least two sites with ≥5 mm pocket depth with ≥2 mm clinical attachment loss and radiographic bone loss on first molars and/or incisors, but on no more than two teeth other than first molars and incisors [1]. Periodontally healthy individuals were also 5–25 years of age, and presented with no pocket depth ≥5 mm and absence of clinical attachment loss by full mouth probing and no evidence of bone loss by radiographic imaging. All LAP patients had a history of site progression based on radiographic assessment, attachment loss of ≥3 mm, no more than two teeth affected by bone loss other than first molar and incisor, and had not yet received periodontal treatment. Patients were excluded based on: diagnosis with systemic diseases or conditions, or currently taking medications that could influence the progression and/or clinical characteristics of periodontal diseases; having taken antibiotics within the last 3 months; tobacco use; pregnant/lactating; or having received active periodontal treatment within the past 6 months. This study included 14 untreated LAP patients, 15 healthy siblings of LAP patients, and 13 unrelated healthy controls (Table 1).

**Table 1.** Patient demographics

	LAP	Healthy sibling (HS)	Healthy control (HC)
Age (years) ±*SD*^a^	13.9 ± 4.8	11.2 ± 3.3	19.5 ± 5.6
Sex			
Male	6	8	5
Female	8	7	8
Total	14	15	13
Ethnicity			
African-American	14	15	11
Caucasian	0	0	2

^a^No difference noted among groups for age, sex, or ethnicity distribution.

LAP, localized aggressive periodontitis.

### Patient PBMC isolation

**Figure 1.**
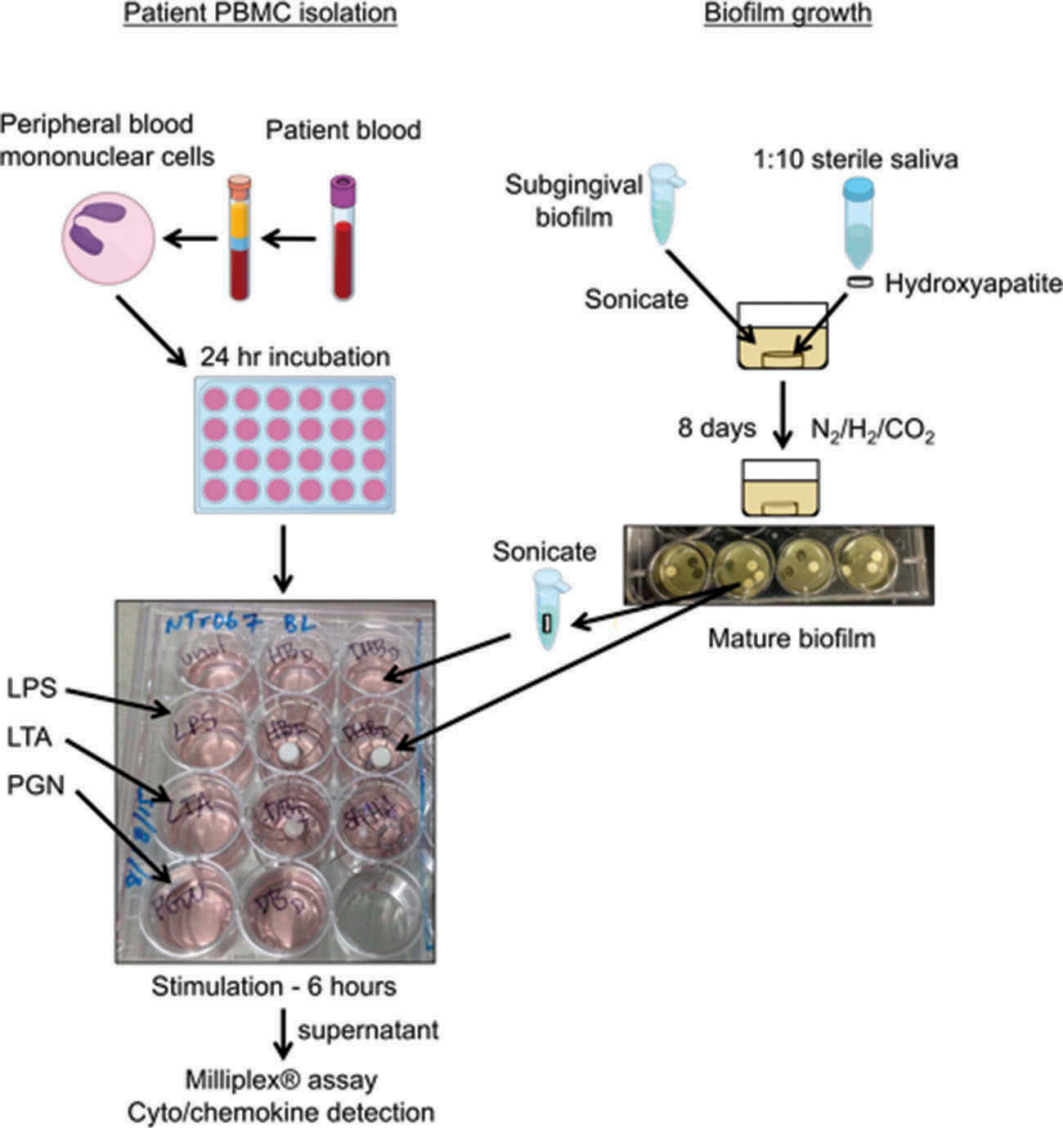
Flow chart of experimental methods including biofilm growth, patient peripheral blood mononuclear cell (PBMC) isolation, and PBMC stimulation with bacterial cell surface components, intact, and disbursed biofilms.

Patient whole blood was collected and stored overnight at 4°C. Samples were divided for analysis and part sent for leukocyte population assessment by flow cytometry, which demonstrated equivalent numbers of mononuclear cells as well as lymphocytes between individual patients (data not shown). PBMCs were isolated using a Ficol gradient following the manufacturer’s instructions. Patient PBMCs were suspended at a final concentration of 5 × 10^4^ cells/mL in RPMI-1640 complete (RPMI-1640, 5% fetal bovine serum, 1% P/S/A, 1% sodium pyruvate, 1% HEPES, 0.01% β-mercaptoethanol), and 5 × 10^4^ cells per stimulant were incubated at 37°C/5% CO_2_ overnight to rest before being stimulated. Figure 1 presents a flow chart of the methods for patient PBMC isolation, biofilm growth, and PBMC stimulation.

### *In vitro* growth and perpetuation of biofilms

Whole subgingival biofilm plaque samples were obtained from one LAP patient diseased site, a healthy site from the same LAP patient, and from a healthy unrelated control healthy site, designated DD, DH and HH, respectively, as previously described [25], and an unstimulated saliva sample was collected from the same patients. Biofilms were grown, as previously described [23], for stimulations on sterile saliva-coated hydroxyapatite disks. This method was shown to maintain similar microbial community composition throughout seven generations [25]. Biofilms were grown in anerobic conditions, and media was replaced every 2 days. Biofilms were grown 8 days to maturation, and then one disk with biofilm was removed, sonicated in Ringer solution, and used to inoculate a new generation on freshly saliva-coated disks in tryptic soy broth supplemented with 5 µg/mL of hemin and 1 µg/mL of menadione. Biofilms were grown for 10 generations before new subgingival biofilm plaque samples were collected and the procedure repeated.

### Preparation of biofilms for PBMC stimulation

Complex whole biofilms were grown as described for 8–9 days and prepared for stimulation as either intact biofilm or dispersed biofilm. Intact biofilms were used as they were, attached to the hydroxyapatite disk and undisturbed, briefly dipped into sterile Ringer solution to remove non-adherent bacteria, while dispersed biofilms were prepared as follows: one whole disk was removed from the culture well, briefly dipped into sterile Ringer solution to remove non-adherent bacteria and placed in 1 mL Ringer solution, sonicated for 10 s, diluted based on growth-curve analyses into RPMI-complete, and aliquoted into the appropriate well at 1 × 10^4^ bacterial cells/mL. This concentration was decided by a pilot study that determined 10^4^ bacterial cells/mL provide sufficient stimulation to elicit cytokine release within the range of detection (unpublished results).

### Stimulation of patient PBMCs

Patient PBMCs that had rested overnight were stimulated with the following: *Escherichia coli* LPS (O111:B4 UltraPure, 5 µg/mL), *Staphylococcus aureus* LTA (5 µg/mL), and *S. aureus* PGN (5 µg/mL; all from Invivogen, San Diego, CA); intact biofilm HH, DH, and DD, one disk each prepared as above and placed inverted on a stainless-steel suspender so that the biofilm was in contact with the PBMC suspension. Dispersed biofilms HH, DH, and DD were prepared as above, and 1 × 10^4^ cells/mL was added to the appropriate wells. The PBMCs were incubated at 37°C/5% CO_2_ for 6 h, then the cell culture media was collected and spun for 10 min at 5,000 × g to pellet PBMCs and bacterial cells, and the supernatant was aliquotted and frozen at −80°C for cytokine analysis. One control well contained only the stainless-steel suspender and a sterile hydroxyapatite disk, while the second control well had nothing added to the PBMCs. At the time of collection, all samples that had been exposed to bacteria had Complete Mini protease inhibitor cocktail (Roche, Indianapolis, IN) added to prevent further degradation of cytokines by bacterial proteases. Post stimulation, all wells were visually inspected at 20×; magnification for PBMC viability, and the overwhelming majority appeared ‘bright,’ indicating that cell death during stimulation was minimal and similar among groups.

### Cytokine analysis

Secreted cyto/chemokine concentration was determined by Milliplex Assay (Millipore, St. Charles, MO) that detected GM-CSF, Eotaxin, interferon gamma (IFN-γ), IL-1β, IL-2, IL-6, IL-8, IL-10, IL-12p40, IL-12p70, IP10, MCP-1, MIP-1α, and tumor necrosis factor alpha (TNF-α). Supernatant samples were thawed on ice, and the Milliplex assay was performed following the manufacturer’s directions. Samples were read using Luminex® 200™ and analyzed with Milliplex Analyst software (Millipore) with five parameter logistics and standard curves. Cyto/chemokines Eotaxin, IFN-γ, IL-2, IL-12p70, and IP10 were below the detection limit and were excluded from analyses. Fold change was determined by dividing the cytokine concentration of stimulated PBMCs for every patient by the corresponding unstimulated PBMC cytokine value. For intact biofilm stimulations, the average cytokine concentration was divided by the concentration from PBMCs presented with a sterile hydroxyapatite disk on a stainless-steel suspender. The average fold change in cytokine concentration from unstimulated PBMCs in each patient group for each cytokine and stimulant is presented in Supplementary Table S1, ±SE.

### Data reduction: principal component analysis

Principal components analysis (PCA) was conducted to identify principal components of immune response on cytokine concentrations for each patient sample using varimax rotation. Components with eigenvalues >1 were retained for interpretation and confirmed by visual inspection of the scree plot. PCA uses interrelations among variables to form a smaller number of components.

### Discriminant function analyses

Each of the principal components above were then subjected to discriminant function analysis, which generated one or more functions to describe the variations in the data, and generated plots with group centroids if more than one function was defined. The principal component showing best separation of the group centroids (indicated by black dots in PCA figures) was selected for further analysis. Significance of separation of group centroids was determined for each discriminant analysis by chi-square test or Wilks’ lambda for all functions generated in each discriminant analysis, and *p*-values of ≤0.05 were considered significant.

### Statistical analysis

Significant differences in cytokine concentrations between patient groups and between stimulants were determined by analysis of variance (ANOVA) using GraphPad Prism v5 (GraphPad Software, Inc., San Diego, CA), and *p*-values ≤0.05 were considered significant. While ANOVA of patient group ages demonstrated a significant difference between HS and HC, multivariate analysis of variance (using IBM SPSS Statistics for Windows v23; IBM Corp., Armonk, NY) of patient groups controlling for age determined that there was no significant difference in the data for each stimulation for all cytokines related to age differences. IBM SPSS Statistics for Windows v23 was used to perform the principal component analysis and the discriminant function analysis as described above.

## Results

### LPS stimulation elicits more robust cyto/chemokine responses in PBMCs than LTA or PGN

As LAP patient whole blood is known to exhibit a hyper-response to LPS stimulation, the study first assessed whether LAP patient PBMCs likewise exhibited a hyper-response to LPS stimulation. It was found that LAP patient PBMCs stimulated with LPS did not demonstrate a hyper-responsive phenotype compared to healthy control responses for any of the individual cyto/chemokines measured (Supplementary Table 1). The average cytokine response was slightly higher in LAP than in HC PBMCs for only IL-12p40 and MCP-1, while the remaining cytokines were equivalently induced in PMBCs from HC and LAP patients (Supplementary Table 1). Healthy sibling PBMC responses were slightly lower than LAP PBMC responses in all cyto/chemokines measured, similar to a previous report using whole-blood stimulation [3] (Supplementary Table 1).

Additionally, the study examined the response of PBMCs to the bacterial surface components LTA and PGN in order to determine if LAP patient PBMCs exhibit a hyper-response to bacterial molecules other than LPS. Stimulation with either LTA or PGN did not elicit a hyper-response in LAP PBMCs compared to HCs (Supplementary Table 1). In addition, PBMCs from HS exhibited the lowest average cytokine expression following LTA or PGN stimulation. Interestingly, both stimulants induced an overall lower average cytokine response than LPS stimulation, regardless of patient population (Supplementary Table 1). Specifically, LAP patient PBMC responses to LPS stimulation were significantly higher than to LTA stimulation for IL-1β, IL-12p40, MIP-1α (*p* ≤ 0.05) and IL-6 and TNF-α (*p* ≤ 0.01), and to PGN stimulation for IL-12p40 (*p* ≤ 0.05; Supplementary Table 1). HS PBMC responses to LTA and PGN were significantly lower than to LPS for IL-6 and TNF-α (*p* ≤ 0.01; Supplementary Table 1). Together, these data indicate that while an immune hyper-responsiveness was not observed in the LAP patient population, LPS appears to be the most potent stimulant tested in these patients and their susceptible siblings.

Interestingly, a bimodal response pattern was observed following LPS, LTA,and PGN for all cytokines measured in the LAP and HS cohorts, which we have termed HI (high) and LO (low) responders. Of the 14 LAP patients, seven were HI responders in at least 50% of the stimulations, while of the 15 HS patients, four were HI responders in at least 50% of the stimulations. While HI responder patients were nearly universally HI responders to LPS for the cyto/chemokines evaluated, the same patients were often in LO responders following LTA and PGN stimulations for the same cyto/chemokines, with the exception of MCP-1.

### Conditions of health and disease cannot be discriminated by response to bacterial surface components

**Figure 2.**
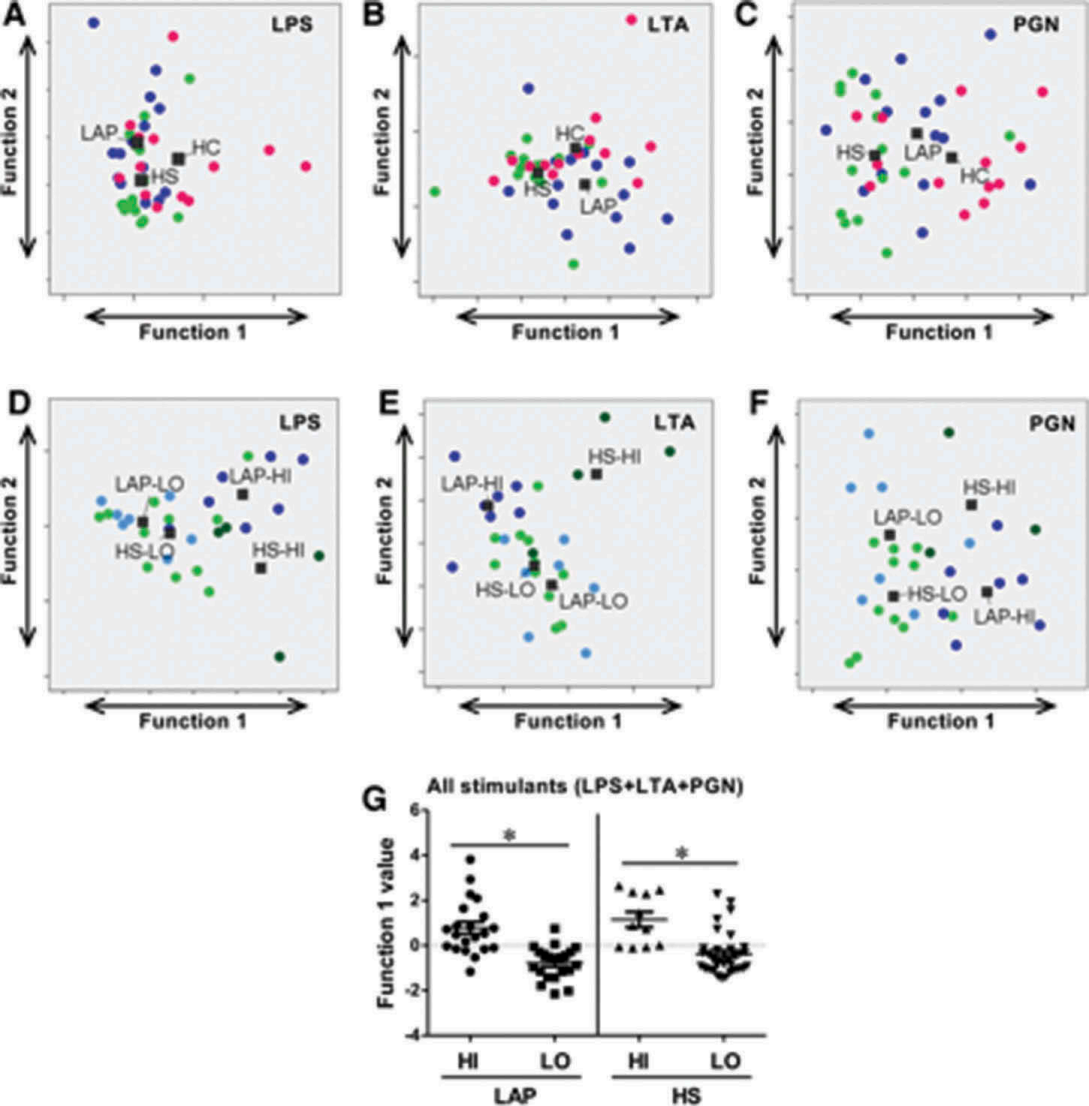
PBMC responses to different bacterial surface components can discriminate within but not between patient groups. Discriminant analysis of localized aggressive periodontitis (LAP), healthy sibling (HS), and healthy unrelated control (HC) PBMC responses to (**A**) lipopolysaccharide (LPS) stimulation, (**B**) lipoteichoic acid (LTA) stimulation, and (**C**) peptidoglycan (PGN) stimulation cannot significantly separate patient groups. Discriminant analysis of LAP hight (HI)/low (LO) and HS HI/LO PBMC responses to (**D**) LPS stimulation, (**E**) LTA stimulation, and (**F**) PGN stimulation; only LTA and PGN discriminations are significant among groups. (**G**) Both LAP and HS HI and LO responder groups can be significantly discriminated by responses to LPS, LTA, and PGN combined. Discriminant analysis statistical values are presented in Table 2. Each dot indicates one patient. (**A–C**): blue dots, LAP; green dots, HS; pink dots, HC. (**D–F**): dark blue dots, LAP-HI; light blue dots, LAP-LO; dark green dots, HS-HI; light green dots, HS-LO. *p ≤ 0.05.

Although the magnitude of the cytokine response to LPS, LTA, and PGN was largely not significant between patient groups, the study aimed to determine if the pattern of cytokine expression within patient groups was significantly different. Thus, PCA was used to determine the groups of cytokines with shared expression patterns to each stimulant. These components were applied to discriminant analyses to identify the group of cytokines, or principal components, that best distinguished the response of LAP, HS, and HC patients within each stimulant. Different principal components best discriminated the patient groups for each stimulant: LPS (IL-1β, IL-6, IL-8, MIP-1α, TNF-α; Figure 2a); LTA (GM-CSF, IL-1β, IL-6, IL-8, IL-10, MIP-1α, TNF-α; Figure 2b); and PGN (GM-CSF, IL-1β, IL-6, IL-8, IL-12p40, MIP-1α; Figure 2c). However, the discrimination of cohorts was not significant (Table 2).

**Table 2.** Bacterial surface component stimulation discriminant analysis Wilks’ lambda and chi-square test of significance

Stimulation	Discrimination	Wilks’ Λ	χ^2^	*df*	*p*
LPS	LAP/HS/HC	0.694	13.50	10	0.197
LTA	LAP/HS/HC	0.693	13.22	14	0.509
PGN	LAP/HS/HC	0.694	12.95	12	0.373
LPS	LAP-HI-LO/HS-HI-LO	0.247	31.46	21	0.066
LTA	LAP-HI-LO/HS-HI-LO	0.17	39.89	21	**0.008**
PGN	LAP-HI-LO/HS-HI-LO	0.213	33.24	21	**0.044**
LPS + LTA + PGN	LAP-HI/LAP-LO	0.609	18.12	7	**0.011**
LPS + LTA + PGN	HS-HI/HS-LO	0.685	14.75	6	**0.022**

Statistically significant values are shown in bold.

LPS, lipopolysaccharide; LTA, lipoteichoic acid; PGN, peptidoglycan; HI, high responder; LO, low responder.

**Figure 3.**
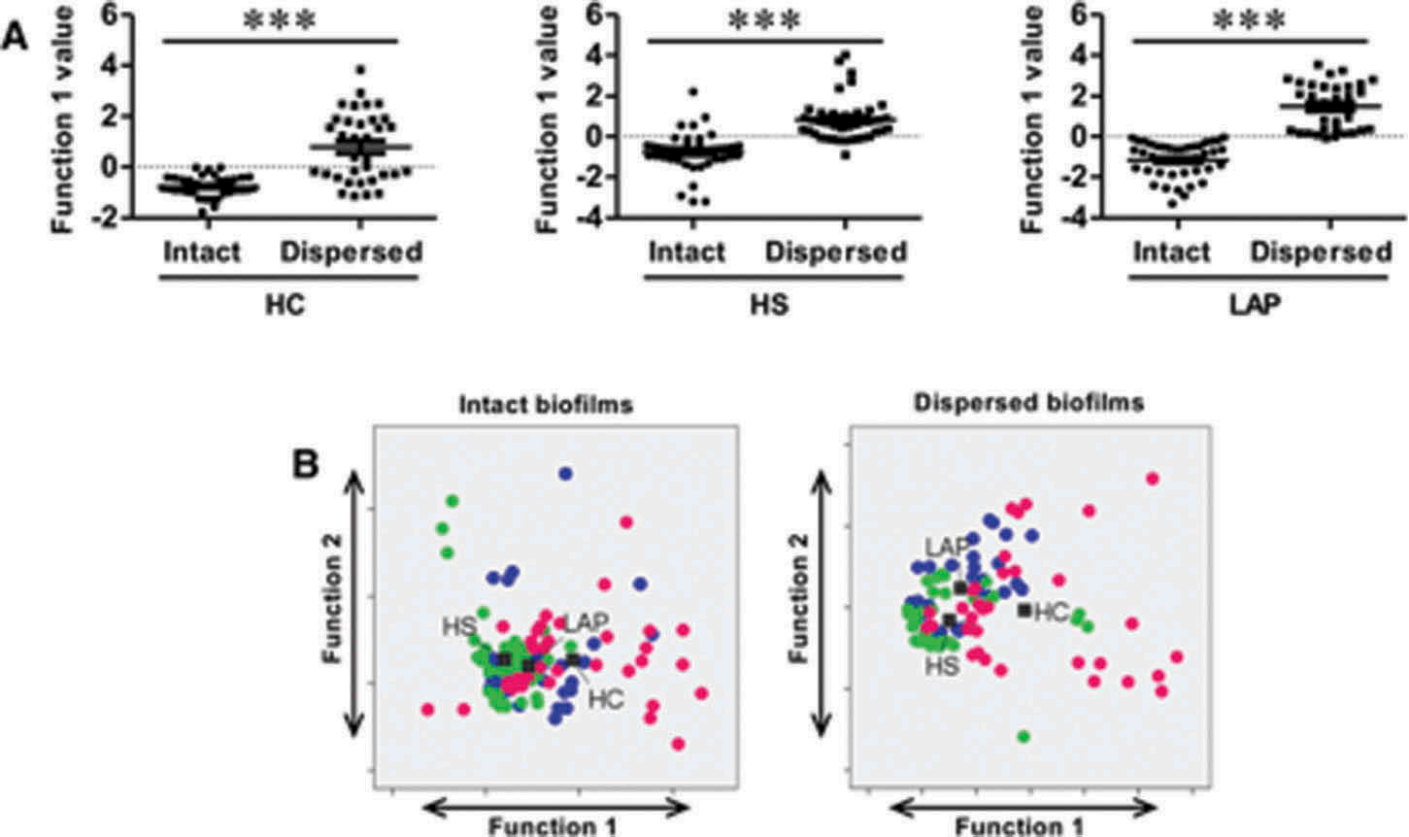
Patient PBMC cyto/chemokine responses discriminate between biofilm state. (**A**) Discriminant analysis significantly separates intact versus dispersed biofilm-stimulated PBMC responses, regardless of source within HC, HS, and LAP groups. (**B**) Combined responses to biofilms of different sources in the same state can discriminate HC, HS, and LAP patient groups for both intact and dispersed biofilms. Discriminant analysis statistical values are presented in Table 3. Each dot indicates a single patient. Blue dots, LAP; green dots, HS; pink dots, HC. ***p ≤ 0.001.

The study also aimed to determine if the PBMC response in the HI and LO responder groups was qualitatively different in both LAP and HS cohorts. Discriminant analysis using cytokine principal components of LAP HI/LO and HS HI/LO response groups determined that the best pattern of discrimination for LPS, LTA, and PGN stimulation was based on expression of GM-CSF, IL-1β, IL-6, IL-8, IL-12p40, MIP-1α, and TNF-α (Figure 2d–f). Importantly, responses to LTA (Figure 3e; *p* ≤ 0.01) and PGN (Figure 3f; *p* ≤ 0.05) were statistically qualitatively different between those two groups (Table 2). Lastly, the qualitative PBMC cytokine responses in HI and LO responders in the LAP and HS cohorts to all LPS, LTA, and PGN stimulations combined were examined in order to determine if there are unique response patterns in the HI and LO patient groups, regardless of type of innate immune stimulation. Discriminant analysis of principal components demonstrated that between HI and LO responders, there is a significant difference in the pattern of cyto/chemokine expression by LAP-derived PBMCs (*p* ≤ 0. 05) based on GM-CSF, IL-1β, IL-6, IL-8, IL-12p40, MIP-1α, and TNF-α, and by HS-derived PBMCs (*p* ≤ 0. 05) based on GM-CSF, IL-1β, IL-6, IL-8, MIP-1α, and TNF-α (Figure 2g and Table 2). Together, the results indicate that investigating the qualitative differences in cytokine responses rather than magnitude of cytokine responses may be more useful to understand differences in not only disease states but also disease susceptibility.

### Magnitude of cyto/chemokine responses to intact and dispersed biofilms suggest PBMC response is dependent on biofilm state

Whether LAP patient PBMCs exhibit a hyper-responsive phenotype to stimulation with intact or dispersed biofilms is not yet known. Thus, LAP-, HS-, and HC-derived PBMCs were stimulated with intact and dispersed biofilms from either diseased or healthy periodontal sites. LAP-derived PBMCs did not exhibit a hyper-response to biofilm, regardless of biofilm state (intact or dispersed) or sources (DD, DH, or HH) for all cytokines examined (Supplementary Table 1) when compared to HS- or HC-derived PBMCs. Again, a bimodal distribution of responsiveness was observed in the LAP and HS cohorts, regardless of biofilm state or sources, resulting in the designation of HI and LO responders, and again, HI responders were defined as those who responded most robustly to at least 50% of all of the stimulations for at least 50% of the cytokines measured.

The average cyto/chemokine responses by LAP-, HS-, and HC-derived PBMCs were equivalent within the same biofilm state, regardless of the biofilm sources, indicating that the magnitude of the response to biofilms may not depend on the microbial community but rather on whether the biofilm is disturbed. Conversely, when measuring the magnitude of the cyto/chemokine responses to either intact or dispersed biofilms, two distinct patterns of reactivity were shared by PBMCs derived from LAP, HS, and HC cohorts. Specifically, GM-CSF, IL-6, IL-8, IL-10, IL-12p40, MCP-1, and MIP-1α were expressed at higher levels in dispersed biofilm stimulations compared to intact biofilm stimulations (Supplementary Table 1), while IL-1β and TNF-α were expressed at the same level or slightly higher in intact biofilm stimulations compared to dispersed biofilm stimulations (Supplementary Table 1). Notably, all average cyto/chemokine responses to dispersed biofilms were equivalent to LPS response values, demonstrating that dispersed biofilms are equally potent at stimulating PBMCs.

### Biofilm state but not source discriminates qualitative differences in immune responses under conditions of periodontal health and disease

To determine whether PBMCs derived from LAP, HS, or HC cohorts respond in qualitatively different manners to different biofilm states (intact vs. dispersed) regardless of source (from healthy or diseased sites), discriminant analyses were performed on principal components from responses to intact biofilms from DD, DH, and HH sites as a whole as compared to dispersed biofilms from DD, DH, and HH sites as a whole within the LAP, HS, and HC cohorts. Factor and discriminant analyses determined that the principal component that best discriminates intact biofilm responses from dispersed biofilm responses differs between LAP (GM-CSF, IL-1β, IL-6, IL-8, MIP-1α, TNF-α), HS (IL-1β, IL-6, IL-8), and HC (IL-6, IL-8, IL-12p40, MIP-1α; Figure 3a), whereby the separation of PBMC responses by each patient population is significant (*p* ≤ 0.001; Table 3). These results indicate that biofilm state is an important driver of the immune response in both periodontally diseased and periodontally healthy cohorts.

**Table 3.** Intact and dispersed subgingival biofilm stimulation discriminant analysis Wilks’ lambda and chi-square test of significance

Group	Discrimination	Wilks’ Λ	χ^2^	*df*	*p*
HC	cB_i_/cB_d_	0.617	31.36	4	**<0.0001**
HS	cB_i_/cB_d_	0.606	41.79	3	**<0.0001**
LAP	cB_i_/cB_d_	0.365	70.57	6	**<0.0001**
HC^a^	HH_i_/DH_i_/DD_i_	0.851	4.92	8	0.766
HS^a^	HH_i_/DH_i_/DD_i_	0.956	1.87	4	0.760
LAP^a^	HH_i_/DH_i_/DD_i_	0.814	7.41	14	0.917
HC^a^	HH_d_/DH_d_/DD_d_	0.96	1.19	8	0.997
HS^a^	HH_d_/DH_d_/DD_d_	0.87	5.07	12	0.955
LAP^a^	HH_d_/DH_d_/DD_d_	0.895	3.04	12	0.995
cB _i_	LAP/HS/HC	0.734	36.36	8	**<0.0001**
cB_d_	LAP/HS/HC	0.651	44.65	10	**<0.0001**

Statistically significant values are shown in bold.

^a^Data not graphed.

cB, combined biofilm sources; i, intact biofilm; d, dispersed biofilm; HH, biofilm from healthy control; DH, biofilm from healthy site in LAP patient; DD, biofilm from LAP disease site.

Next, the study determined whether PBMCs derived from LAP, HS, or HC cohorts respond in qualitatively different manners to different biofilm sources (DD, DH, HH) in order to determine whether the biofilm microbial composition is an important driver in PBMCs responses. Discriminant analyses using principal components of cytokines were performed on LAP-, HS-, and HC-derived PBMC responses to either intact or dispersed biofilm based on whether the biofilm was isolated from either DD, DH, or HH sites. It was found that the PBMC responses to biofilms of all three sources were not significantly qualitatively different in any patient cohort, regardless of whether the biofilm was intact or dispersed (Table 3). These results demonstrate that unlike biofilm state, the biofilm source and thus microbial composition is not an important driver of the immune response in both periodontally diseased and periodontally healthy cohorts.

Since it was found that biofilm state but not source is an important driver of the immune response in PBMCs in healthy and diseased patient groups, the study sought to determine if the PBMC pattern of response overall to intact and dispersed biofilms was qualitatively different in the LAP, HS, and HC cohorts. Thus, discriminant analysis based on principal components was performed on LAP-, HS-, and HC-derived PBMC responses to DD, DH, and HH intact biofilms combined and DD, DH, and HH dispersed biofilms combined, to separate LAP, HS, and HC patient groups. Different principal components were able to significantly discriminate between the three patient groups for both intact (IL-1β, IL-8, MIP-1α, TNF-α; *p* ≤ 0.001; Figure 3b and Table 3) and dispersed (IL-6, IL-8, IL-12p40, MIP-1α, TNF-α; *p* ≤ 0.001; Figure 3b and Table 3) biofilm stimulations. This result suggests that activation of multiple signaling pathways by a complex assortment of immunostimulants distinguishes healthy from periodontally diseased and susceptible patient cohorts where activation of a single pathway by a specific stimulant such as LPS does not.

### PBMC response to LPS is qualitatively different from response to dispersed biofilms in LAP patients but not healthy individuals

**Figure 4.**
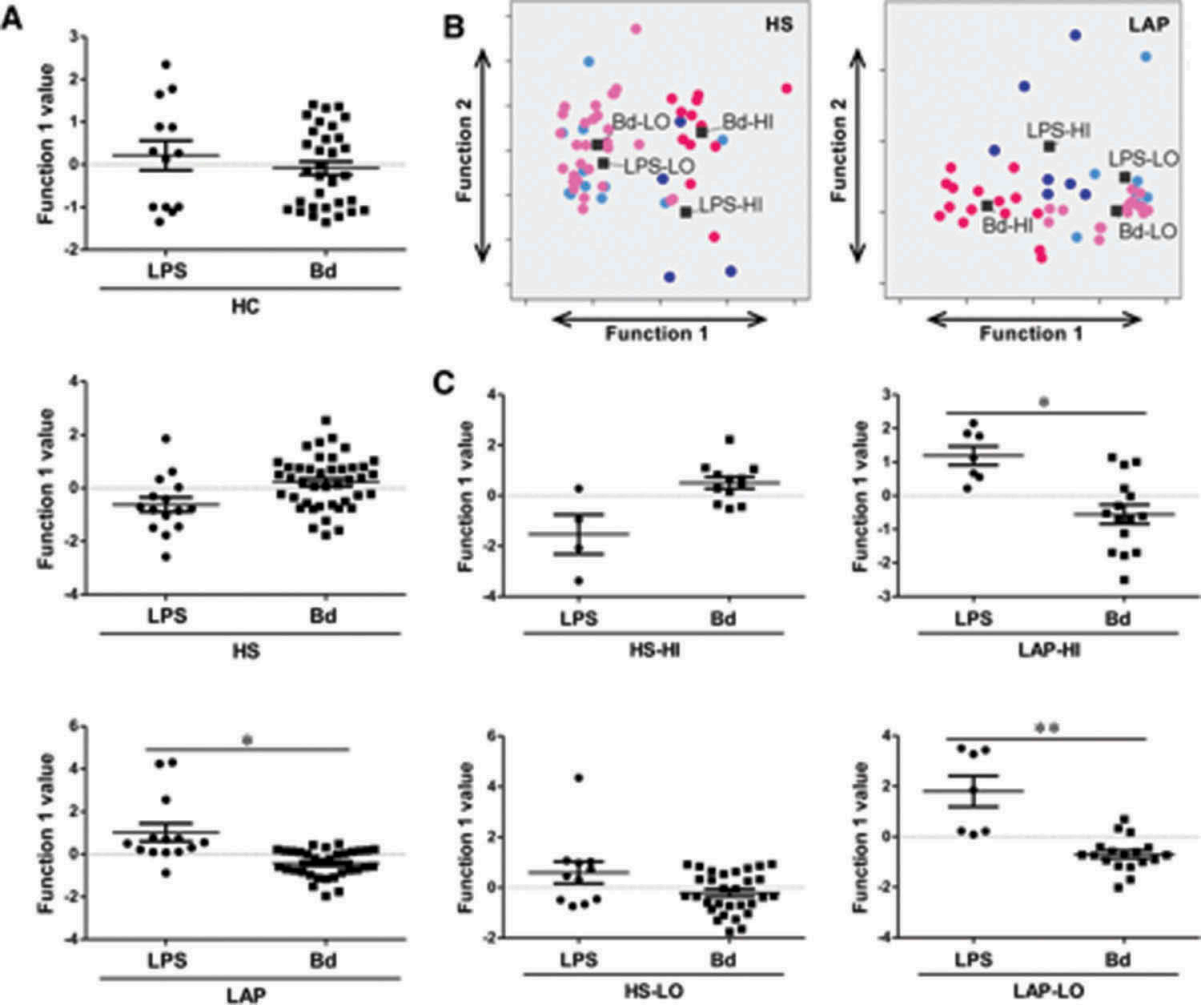
LAP but not HS or HC PBMCs discriminate responses to LPS and dispersed biofilm. (**A**) Discriminant analysis shows that only LAP patients present a different pattern of response when stimulated with LPS versus dispersed biofilm. (**B**) HS and LAP HI/LO response groups separate significantly by discriminant analysis. (**C**) Individual discriminant analyses of HS and LAP HI and LO responder groups to LPS and dispersed biofilm shows a significant difference in response pattern in LAP PBMCs only. Discriminant analysis statistical values are presented in Table 4. Each dot indicates a single patient. Bd, dispersed biofilm combined responses from HH, DH, and DD stimulations. Dark blue dots, LPS-HI; light blue dots, LPS-LO; dark pink dots, Bd-HI; light pink dots, Bd-LO. *p ≤ 0.05; **p ≤ 0.01.

The magnitude of the average cyto/chemokine response to LPS and dispersed biofilms from all three sources was equivalent for all nine cyto/chemokines measured (Supplementary Table 1), demonstrating that dispersed biofilms are just as potent as LPS at stimulating PBMC responses. Complex oral biofilms contain many potential inducers of inflammation in addition to LPS that are released when the biofilms are disrupted. Thus, in order to indicate how influential the LPS component of the biofilm was in driving the PBMC response to dispersed biofilms, discriminant analyses of LAP-, HS-, and HC-derived PBMC responses to LPS as it compared to dispersed biofilm responses were run. Here, it was determined that the principal component that best separated LPS and dispersed biofilm PBMC responses for LAP as well as HS stimulations (GM-CSF, IL-1β, IL-6, IL-8, MIP-1α, TNF-α) differed from the principal component that best separated the HC-derived PBMC responses (IL-10, IL-12p40; Figure 4a). However, only LAP patients show a significantly different pattern of response to LPS versus dispersed biofilm (*p* ≤ 0.05), whereas the healthy individuals show similar pattern of response (Figure 4a and Table 4). As HC- and HS-derived PBMC responses to LPS and dispersed biofilm could not be discriminated, this result suggests that the HC- and HS-derived PBMC responses to dispersed biofilms are more similar to responses to LPS than is the LAP-derived PBMC response to dispersed biofilms. Hence, LAP patient PBMCs appear to respond uniquely to additional stimulants.

**Table 4.** LPS versus dispersed subgingival biofilm stimulation discriminant analysis Wilks’ lambda and chi-square test of significance

Group	Discrimination	Wilks’ Λ	χ^2^	*df*	*p*
HC	LPS/cB_d_	0.982	0.819	2	0.664
HS	LPS/cB_d_	0.874	6.998	6	0.321
LAP	LPS/cB_d_	0.685	15.89	6	**0.014**
HS	LPS-HI/LPS-LO/cB_d_-HI/cB_d_-LO	0.271	66.63	18	**<0.0001**
LAP	LPS-HI/LPS-LO/cB_d_-HI/cB_d_-LO	0.146	78.92	18	**<0.0001**
HS-HI	LPS/cB_d_	0.528	7.663	4	0.105
HS-LO	LPS/cB_d_	0.881	4.496	7	0.721
LAP-HI	LPS/cB_d_	0.579	9.844	4	**0.043**
LAP-LO	LPS/cB_d_	0.421	17.735	5	**0.003**

Statistically significant values are shown in bold.

Similarly, discriminate analyses of principal components were applied in order to determine if the PBMCs from HI and LO responders qualitatively differed in their response to LPS and dispersed biofilms, independent of patient disease status (i.e. LAP or HS). Here, the same principal component best discriminated between HI- and LO-responder PBMC responses to LPS as well as dispersed biofilm responses, regardless of patient cohort (GM-CSF, IL-1β, IL-6, IL-8, MIP-1α, TNF-α; Figure 4b). Discriminate analysis revealed that regardless of patient cohort, both HI and LO responders have statistically different qualitative responses to LPS than dispersed biofilms (*p* ≤ 0.001; Figure 4band Table 4).

In addition, in order to determine if disease status drove the PBMC response to LPS and dispersed biofilms in HI and LO responders, discriminant analysis was used to determine if the PBMCs from LAP and HS HI and LO responders individually qualitatively differed in their response to LPS and dispersed biofilms. Here, there was not a qualitative difference in the way HS HI- (IL-10, IL-12p40, MCP-1, MIP-1α) or LO-responder (GM-CSF, IL-6, IL-8, IL-12p40, MCP-1, MIP-1α, TNF-α)–derived PMBCs responded to LPS or dispersed biofilm responses (Figure 4c, left panels). Conversely, there was a statistically significant qualitative difference in the way both LAP HI- (IL-1β, IL-6, MIP-1α, TNF-α) and LAP LO-responder (IL-8, IL-10, IL-12p40, MCP-1, TNF-α)–derived PBMCs responded to LPS and dispersed biofilms (*p* ≤ 0.05 and *p* ≤ 0.01, respectively; Figure 4c, right panels). These data indicate that periodontal disease status is also a driver of the PBMC response to LPS and dispersed biofilms. Taken together, these data indicate that LAP response to biofilms is indeed unique and that within this group as well as their susceptible siblings, there are different individual responders (LO and HI responders), who may have different innate sensing mechanisms that contribute to disease development and progression.

## Discussion

Much of the focus on pathogenesis of LAP has been on altered neutrophil function [26], suggesting that inefficient chemotaxis, poor activation, or failure to resolve inflammation prevent an appropriate response to subgingival microflora and promote tissue destruction [27]. Here, evidence is presented that PBMCs of LAP patients respond to stimulation with different bacterial components or biofilms by unique patterns of cytokine expression, which could enhance and/or promote disease progression in myriad ways. In particular, there appear to be two different patient populations based on differences in the magnitude of cytokine responses to bacterial stimulations. Yet, these groups also differ in their cytokine expression patterns, which supports that there are multiple complex mechanisms responsible for development of LAP.

The bimodal response patterns in LAP and HS PBMCs, which we have separated into HI and LO responders, demonstrate the importance of assessing individual patient responses. Patients sharing the same diagnosis may respond differently to different stimuli, such that the mechanism of disease development and/or progression differs between individuals, while the clinical manifestations of disease appear the same. This may be due to individual sensitivity to specific immunostimulants, or propensity to respond more robustly to specific immunostimulants, based in differences in genetics [28]. For example, several single nucleotide polymorphisms have been associated with LAP [29], as have differences in epigenetics [30], although these are not found within all LAP patients. Additionally, LAP destruction occurs in bursts of activity between periods of quiescence [31], and immune cells collected from a patient experiencing active disease may respond differently from cells collected from a patient during a quiescent period. As the blood collected for this study was from patients attending an initial periodontal examination, it is not known if any were experiencing active tissue destruction. However, this can be determined in the future by clinical metrics when patients attend follow-up appointments, and these studies are in progress. In future studies, it will be of interest to determine if the cytokine response patterns of PBMCs can discriminate patients experiencing active versus quiescent disease, or if patterns are representative of patients whose disease has stabilized compared to those whose disease will continue to progress. Therefore, within the healthy sibling group, the intention is to follow the HI and LO responders to determine if either is predictive of developing LAP. Additionally, two recent reports on immune response–related gene expression patterns demonstrate that individual ancestry plays a significant role in how certain cells respond to immune stimulants [18,32]. Notably, individuals of African and European ancestry have distinct monocytic cell transcriptional responses to specific TLR stimulants, whole bacterial, or whole viral infection. However, the PBMC responses of the few Caucasian control patients in this study were not outliers regarding any measurements that were taken, compared to the African American controls, and they have been left in the analysis because the groups were very small. In the future, it will be important to include only African American controls in order to avoid obscuring significant results.

A major difference in the findings compared to previous reports using whole-blood stimulations is that the PBMCs did not exhibit a hyper-inflammatory response to stimulation with LPS or any of the stimulants used. While this was unexpected, it provides insight into the differences between the potential of cells to respond to stimulation and the actual response in a natural environmental milieu. Whole blood contains circulating cyto/chemokines as well as circulating stimulants such as LPS [33] that may enhance responses to exogenously added stimulants, which could result in a hyper-inflammatory response, and is representative of a patient’s current inflammatory status. Ficol-purified PBMCs have all the additional serum components removed, and therefore responses to exogenously added stimulants represent the potential in any one individual to respond to stimulation within a given time frame. However, the observed separation of LAP patients and their healthy siblings into HI and LO responders based on magnitude of PBMC cyto/chemokine response mirrors a similar separation seen in whole blood [34], suggesting that a difference in the potential to respond is actualized in a natural setting. One focus of future studies will be to compare the differences in responses to stimulation of whole blood and PBMCs isolated from the same patient. This comparison will demonstrate whether a patient PBMC population has an underlying propensity for a high response to stimulation, as well as whether whole blood does indeed show a more robust response, possibly due to the presence of different stimulants and cells. In addition, the samples were stimulated for a shorter time, 6 h rather than 24 h, which may also be a reason for the lack of difference in magnitude of cyto/chemokine response between LAP patients, their healthy siblings, and the unrelated healthy controls, as observed in whole blood in the past [3]. Future studies will also compare 6 h versus 24 h stimulations in order to understand the extent to which exposure time influences the observed cyto/chemokine response in these individuals.

While it is common to use a single immunostimulant such as LPS to study immunological responses, the present data demonstrate that responses to individual bacterial components, specifically LPS, LTA, and PGN, cannot be used to discriminate patient groups, whereas responses to whole biofilms can. Although LPS is a potent stimulant of the host response and important for evaluating differentiated responses in different individuals, whole biofilm interaction with the host may be more insightful than single stimulants, as it mimics the bacterial components in the periodontal pocket and their collective influence on the host. It may be that biofilm as a whole present a complex array of stimulants that trigger multiple receptors in the cells, providing a different pattern of response and therefore better discrimination. Comparing patterns of responses to LPS versus dispersed biofilms demonstrated that although the robustness of the PBMC response appears identical, there are distinct differences in the way they are responding. The data collectively suggest that studying responses to single stimulants alone cannot explain mechanistic differences in immunological responses between patient groups, and highlight the complexity of the immune response in individuals experiencing chronic inflammation.

Distinct differences were observed in the responses to intact versus dispersed biofilms regarding both the magnitude of the cytokine response and the pattern of the cytokine response, which are supported by other published studies. Specifically, different strains of *E. coli* grown as biofilm then mechanically disrupted were poorly phagocytized by PBMC-matured naïve macrophages compared to planktonic *E. coli* [22], and biofilm-grown *Staphylococcus epidermidis* elicited reduced activation of nuclear factor kappaB compared to isogenic biofilm-negative strains [24]. Additionally, Thurlow et al. [35] reported that macrophages are skewed to an M2 phenotype when exposed to *S. aureus* in a biofilm, rendering them less inflammatory and less capable of clearing the biofilm than M1-type macrophages [36]. One explanation for this observation is that intact biofilms are less recognizable and more difficult to phagocytose by macrophages due to the extracellular matrix that coats and ‘hides’ the bacteria in the biofilm [35], so there are a limited number of immunostimulants for innate immune recognition. Mechanical disruption of the biofilm breaks up the matrix and exposes numerous additional bacterial products that were previously secluded from immune recognition, which elicit a more robust macrophage response.

The discriminant analyses revealed that PBMCs, whether derived from a healthy or a diseased background, distinguish between intact biofilms and dispersed biofilms in their cyto/chemokine response patterns. Yet, the PBMCs do not discriminate between the biofilms derived from healthy and diseased periodontal sites. Therefore, it appears that biofilm source (i.e. derived from a healthy or diseased site) does not influence pattern of immune response, while biofilm state (i.e. homeostatic vs. disequilibrium) does influence the pattern of immune response. This has implications for understanding development and progression, not just in periodontal diseases but for all biofilm-mediated infections. These results suggest that biofilm from a healthy or a diseased site may not be driving the change in the immune response that leads to tissue destruction. Rather, this change may be driven by biofilm switch from a homeostatic to a disequilibrium state, which ultimately results in changes in the biofilm composition. The results further support evidence presented by Bartold and Van Dyke [37] that microbial differences at periodontal disease sites versus healthy sites are a result of changes in host status that alter the local environment and favor growth of organisms classically considered periodontal pathogens. In conclusion, future studies of biofilm–immune cell interactions will need to consider patient inflammatory state as well as the homeostasis (activity level) of the biofilm to create conditions that best represent those found in patients.

## Supplementary Material

Supplementary_Table_1__1_.docx

## Supplemental material

The supplemental material for this article can be accessed here.
