# Supplementary material for: Cytokine response patterns to complex biofilms by mononuclear cells discriminate patient disease status and biofilm dysbiosis
Source: J Oral Microbiol. 2017 Jun 12;9(1):1330645. doi: 10.1080/20002297.2017.1330645 (PMC5508357; doi:10.1080/20002297.2017.1330645)
Supplement: Supplementary_Table_1__1_.docx [file zjom_a_1330645_sm7908.docx]

Supplementary Table I. Average fold-change in cytokine concentrations for all stimulations of each patient group.

|  |  | Average fold-change in cytokine concentration over unstimulated PBMCs ± SE | | | | | | | | |
| --- | --- | --- | --- | --- | --- | --- | --- | --- | --- | --- |
| Stimulant | Patient group | GM-CSF | IL-1β | IL-6 | IL-8 | IL-10 | IL-12p40 | MCP-1 | MIP-1α | TNFα |
| LPS | LAP | 3.0±1.3 | 89±35 | 73±26 | 12±4 | 6.6±2.4 | 4.51.2 | 3.1±1.0 | 15±5.8 | 78±28 |
|  | HS | 3.0±1.3 | 67±37 | 60±34 | 9±2.7^d^ | 9.6±3.7 | 5.2±3.4 | 1.9±0.58 | 19±5.7 | 58±29 |
|  | HC | 1.8±0.03 | 54±17 | 95±27 | 36±13 | 8.6±3 | 4.4±1.4 | 1.6±0.21 | 98±36 | 88±23 |
| LTA | LAP | 1.0±0.03 | 1.2±0.60^a, g^ | 3.3±0.80^a, h^ | 2.7±0.57 | 1.3±0.24 | 1.2±0.11^c, h^ | 1.4±0.13 | 3.7±1.3^a, g^ | 3.5±0.83^b, g^ |
|  | HS | 1.1±0.05 | 1.6±0.30 | 2.9±0.75^h^ | 2.7±0.39^g^ | 1.3±0.11^g^ | 1.5±0.27^g^ | 1.4±0.11 | 4.3±0.91^g^ | 3.70.84^h^ |
|  | HC | 1.0±0.02 | 1.1±0.05 | 1.9±0.37^i^ | 4.7±1.2^i^ | 1.2±0.09 | 1.3±0.16 | 1.3±0.099 | 9.9±3.1^i^ | 2.8±0.76^i^ |
| PGN | LAP | 1.4±0.23 | 4.3±1.3 | 7.4±2.3 | 3±0.67^h^ | 2.6±1.2 | 1.6±0.30^a^ | 1.3±0.1 | 4.8±2 | 13±4.6 |
|  | HS | 1.2±0.11 | 4.0±1.8 | 5.6±1.6^h^ | 3.1±0.51^d, g^ | 3.0±1.4 | 1.3±0.14^g^ | 1.4±0.1 | 5.7±1.3^g^ | 12±4.4 |
|  | HC | 1.2±0.06 | 4.3±0.98 | 9.2±2.8^h^ | 9.8±3.5^g^ | 2.7±0.98 | 1.3±0.14 | 1.5±0.21 | 24±9.1^g^ | 24±6.7^l^ |
| HHi | LAP | 1.1±0.09 | 11±5.9^n^ | 1.0±0.03 | 1±0^h, p^ | 1±0.014^g, p^ | 1.3±0.26^g^ | 1.0±0.01 | 1.0±0.034 | 1.9±.29^l^ |
|  | HS | 1.2±0.10 | 15±7.7^d, l, o^ | 2.2±1.1^l^ | 1.2±0.20^d^ | 1.7±0.71^h, q^ | 1.0±0.029^h, p^ | 1.0±0.0045 | 1.5±0.46 | 3.5±1.6^l, n^ |
|  | HC | 1.2±0.10 | 3.4±0.8^l, o, p^ | 1.0±0.02 | 1±0.01^g, p^ | 1±0 | 1.1±0.099^g^ | 1.0±0.0018 | 1.0±0.0014 | 1.6±0.17^l, m^ |
| DHi | LAP | 1.3±0.17 | 19±11^d, m^ | 41±21^g, q^ | 7±2.7^d, h, p^ | 7.7±6.2^g, p^ | 2.1±0.41 | 1.4±0.21 | 14±8.5^h, q^ | 43±22^l^ |
|  | HS | 1.9±0.75 | 34±20^f, l, n^ | 37±14^k^ | 8.2±1.9^d, g, p^ | 7.5±2.9^h, p^ | 1.9±0.48^g^ | 1.3±0.11 | 20±6.4 | 46±15^l, m^ |
|  | HC | 1.7±0.48^j^ | 33±11^p^ | 83±27^l, n^ | 24±7.2^k, p^ | 5.6±1.8 | 3.4±1.30^g^ | 1.4±0.16 | 72±24^l, m^ | 96±29^l^ |
| DDi | LAP | 1.1±0.08 | 10±5.9^n^ | 1.1±0.04^p^ | 1.0±0^h^ | 1.0±0^g, p^ | 1.3±0.25 | 1.0±0 | 1.0±0.018 | 1.7±0.22^l^ |
|  | HS | 1.1±0.05 | 11±4.4^d, l, o^ | 1.6±0.61^j, l^ | 1.1±0.15 | 1.1±0.13^g, p^ | 1.1±0.047 | 1.0±0 | 1.3±0.31 | 2.5±0.92^l, n^ |
|  | HC | 1.1±0.12 | 3.8±1.0^g, l, o^ | 1.0±0 | 1.0±0^g^ | 1.0±0^p^ | 1.1±0.053 | 1.0±0 | 1.0±0 | 1.5±0.14^l, m^ |
| HHd | LAP | 1.3±0.20 | 25±15 | 45±24^k, m^ | 6.9±2.8 | 10±8.5 | 2.4±0.55^j^ | 1.4±0.15 | 14±8^j^ | 48±25^k^ |
|  | HS | 2.1±0.93 | 35±18 | 33±15^n^ | 6.9±1.5^d, j,m^ | 4.7±1.6^k^ | 1.5±0.33 | 1.2±0.091 | 20±7^k, n^ | 45±17^l, n^ |
|  | HC | 1.8±0.64 | 33±11 | 91±27^l, n^ | 41±18^l, m^ | 6.9±2.1 | 4.5±1.7 | 1.6±0.22 | 92±30^l, n^ | 128±41^l^ |
| DHd | LAP | 1.2±0.18 | 9.4±5 | 1.1±0.06^k, m^ | 1±0 | 1±0.042 | 1.7±0.63^j^ | 1.0±0.03 | 1.1±0.048^j^ | 2.0±0.37^k^ |
|  | HS | 1.1±0.05 | 7.9±2.8^j^ | 2.7±1.7^d, n^ | 1.3±0.27^e^ | 3.8±2.8^j^ | 1.4±0.26 | 1.0±0.027 | 1.4±0.31^d, j^ | 2.6±0.79^k^ |
|  | HC | 1.1±0.06 | 3.6±0.83^l^ | 1.0±0 | 1±0^g^ | 1±0.0023^g^ | 1.1±0.09 | 1.0±0 | 1.0±0 | 1.4±0.11^l^ |
| DDd | LAP | 1.4±0.22 | 30±19 | 49±27^k, m^ | 7.6±3.3 | 9.1±7.3 | 2.6±0.66^j^ | 1.4±0.23 | 15±8.5^j^ | 54±29^k^ |
|  | HS | 2.0±0.85 | 33±29 | 37±13^o^ | 8.3±2.1^d, j,m^ | 8.8±4.1^j^ | 2.0±0.45^j^ | 1.3±0.014 | 20±6.7^d, k, m^ | 46±16^l, n^ |
|  | HC | 2.4±0.93 | 47±17 | 102±34^l, n^ | 37±15^l, m^ | 7.7±2.8^j^ | 4.5±1.5 | 1.4±0.15 | 84±28^l, n^ | 121±40^l^ |

LPS – lipopolysaccharide, LTA – lipoteichoic acid, PGN – peptidoglycan, HH – healthy patient healthy site biofilm, DH – LAP disease patient healthy site biofilm, DD – LAP disease patient disease site biofilm, i– intact biofilm, d – dispersed biofilm.

^a^ p ≤ 0.05 vs. LPS-LAP same cytokine

^b^ p ≤ 0.01 vs. LPS-LAP same cytokine

^c^ p ≤ 0.001 vs. LPS-LAP same cytokine

^d^ p ≤ 0.05 vs. HC same cytokine, same stimulant

^e^ p ≤ 0.01 vs. HC same cytokine, same stimulant

^f^ p ≤ 0.001 vs. HC same cytokine, same stimulant

^g^ p ≤ 0.05 vs. LPS same cytokine, same patient group

^h^ p ≤ 0.01 vs. LPS same cytokine, same patient group

^i^ p ≤ 0.001 vs. LPS same cytokine, same patient group

^j^ p ≤ 0.05 vs. LTA same cytokine, same patient group

^k^ p ≤ 0.01 vs. LTA same cytokine, same patient group

^l^ p ≤ 0.001 vs. LTA same cytokine, same patient group

^m^ p ≤ 0.05 vs. PGN same cytokine, same patient group

^n^ p ≤ 0.01 vs. PGN same cytokine, same patient group

^o^ p ≤ 0.001 vs. LTA same cytokine, same patient group

^p^ p ≤ 0.05 vs. same source dispersed, same patient group

^q^ p ≤ 0.01 vs. same source dispersed , same patient group
